# Homophilic Interactions of Platelet F11R/JAM-A with its Surface-Bound Counterpart Facilitate Thrombus Formation

**DOI:** 10.1055/a-2565-9496

**Published:** 2025-04-18

**Authors:** Piotr Kamola, Boguslawa Luzak, Patrycja Przygodzka, Cezary Watala, Moro O. Salifu, Anna Babinska, Tomasz Przygodzki

**Affiliations:** 1 Department of Haemostatic Disorders, Medical University of Lodz, Lodz, Poland; 2 Laboratory of Cellular and Molecular Biology, Institute of Medical Biology, Polish Academy of Sciences, Lodz, Poland; 3 Department of Medicine, State University of New York, Downstate Medical Center, Brooklyn, New York, United States

**Keywords:** junctional adhesion molecule-A (F11R/JAM-A), platelets, adhesion, thrombosis

## Abstract

**Background:**

F11Receptor/junctional adhesion molecule-A (F11R/JAM-A) is a transmembrane protein expressed in endothelial cells, epithelial cells, and in blood platelets. In blood platelets, F11R/JAM-A participates in adhesion under static conditions, suppresses the activation of the platelet α _IIb_ β _3_ integrin and was shown to activate blood platelets as soluble form via homophilic interactions.

**Objectives:**

The purpose of presented study was to evaluate whether F11R/JAM-A is involved in platelet adhesion under flow conditions and in thrombus formation.

**Methods:**

F11R/JAM-A contribution to platelet adhesion under flow conditions was assessed using flow chamber assay. Monoclonal antibodies and recombinant F11R/ JAM-A were used to assess the effects of F11R/JAM-A blockade on platelet aggregation and thrombus formation using total thrombus formation analysis system. Effects of F11R/JAM-A blockade on thrombus formation in vivo were evaluated in murine models of carotid artery injury.

**Results:**

F11R/JAM-A was not capable of capturing flowing blood platelets alone but enhanced platelet adhesion to fibrinogen under flow conditions. Blocking of F11R/JAM-A homophilic interactions with specific monoclonal antibodies or with recombinant F11R/JAM-A impaired thrombus formation in vitro in human blood and in vivo in the models of thrombosis in mice.

**Conclusion:**

Interactions of F11R/JAM-A located on flowing platelets with its surface-bound counterpart enhance platelet binding to fibrinogen under high shear stress conditions. Blocking of these homophilic interactions compromises thrombus formation. While previously published studies pointed at a significant role of soluble F11R/JAM-A in priming platelets during thrombus formation, our results highlight the role of surface-bound F11R/JAM-A in this process.

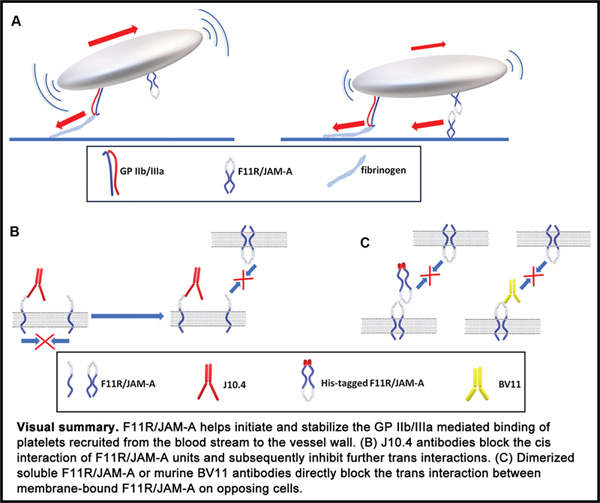

## Introduction

F11Receptor/Junctional adhesion molecule-A (F11R/JAM-A) is a transmembrane protein which belongs to the immunoglobulin superfamily (IgSF). 1 2 The protein was first discovered in blood platelets as a target of an activating F11 antibody. 3 Shortly after, the same protein was identified as a functional component of tight junctions in endothelium and in epithelium. 1 Later, its expression was also detected in immune cells 4 and in smooth muscle cells. 5

The molecule of F11R/JAM-A is composed of an extracellular part, which contains two Ig-like domains, a single-pass transmembrane domain and a short cytoplasmic tail with a PDZ domain-binding motif. 6 7 The extracellular part contains two Ig-like domains, a membrane distal D1 domain and membrane proximal D2 domain. F11R/JAM-A molecules are capable of forming homodimers: in cis-configuration when the dimer is formed by the molecules located in the same cell and in trans-configuration when the interacting molecules are located on membranes of adjacent cells. 8 Both cis- and trans-homophilic interactions are mediated by D1 domain, but the sites responsible for these two types of interactions are located in distinct parts of the molecule, as it was described in detail by Monteiro et al. 8 The only known heterodimeric ligand of F11R/JAM-A is the lymphocyte function-associated antigen (LFA-1) - a leukocyte integrin involved in adhesion and extravasation of lymphocytes. The LFA-1 interacts with JAM-A/F11R via a site located in the proximal D2 domain. 9

A majority of knowledge of the function of F11R/JAM-A comes from epithelial cells, endothelial cells, and lymphocytes, while its role in regulation of blood platelets' activity is still not fully explained, even though the protein was primarily discovered in these elements of blood. 3 10 As a component of tight junctions in endothelial and epithelial cells, the molecules of F11R/JAM-A are assembled as cis-homodimers and interact via trans-homodimerisation with cis-homodimers on adjacent cells. 1 The intracellular part of the protein integrates tight junctions with the actin filaments. Importantly, upon inflammatory activation of endothelial cell, the protein translocates from tight junctions to the apical side of the cell and thus to the luminal surface of the blood vessel facilitating the adhesion of monocytes and platelets to the inflamed vascular wall. 11 12

In blood platelets F11R/JAM-A is associated with αIIbβ _3_ –platelet most dominant integrin. 13 This association, however, is limited to a resting platelet, as platelet activation results in dissociation of the protein from the complex. 14 Murine platelets devoid of the F11R/JAM-A protein not only maintained their ability to aggregate and to form thrombi but were more reactive than their wild-type counterparts. The effect was dependent on Csk kinase. 14 15 This demonstrated that F11R/JAM-A, at least in murine platelets, is not essential to the process of thrombus formation. The activity of platelet F11R/JAM-A, however, is not limited to the regulatory role, as it was shown to form trans-homophilic interactions with its counterpart immobilised on the surface of activated endothelial cells in static conditions. 11 16 Clusters of F11R/JAM-A on contacts between thrombin-activated platelets were identified by electron microscopy. 17 What is more, it was evidenced that a soluble form of F11R/JAM-A increased platelets' ability to form aggregates, which implied that trans-homophilic interactions of this protein play a role during thrombus formation. 18

As can be inferred from the above-mentioned studies, one piece of evidence shows that F11R/JAM-A is not indispensable to thrombus formation, whereas others suggest that trans-homophilic interactions of this protein may occur between platelets. Therefore, it can be speculated that trans-homophilic interactions of F11R/JAM-A play some as of yet undefined role during thrombus formation.

Recent reports pointed to a potential role of platelet F11R/JAM-A in thromboinflammation 18 and drew attention to this new–old player in the process of thrombus formation; hence, we decided to address two specific questions. One of the possible activities of F11R/JAM-A addressed in presented study is the contribution of trans-homophilic interactions of F11R/JAM-A to immobilisation of flowing platelets. Our previous study showed that functional blockade of F11R/JAM-A limited the interaction of flowing platelets with the inflamed vascular wall in vivo. 19 However, the exact contribution of F11R/JAM-A to this process was not elucidated. The second question we addressed was whether the functional blockade of the protein could affect thrombus formation in vitro and in vivo.

## Materials and Methods

### Chemicals

The list of chemicals has been included in the Supplementary Material S1 (available in the online version only).

### Experimental Animals

Mice were housed and bred at the animal facility of the Medical University of Lodz. Food and water were provided ad libitum and mice were housed in groups of up to six animals per cage, under a 12:12 light–dark cycle. Male C57BL/6JRj mice (Janvier Labs, France) were used. Animals included in the experiments were young adults of 8 to 12 weeks, 20 to 24 g. All mouse experimental procedures were approved by the Local Ethical Committee on Animal Experiments, Medical University of Lodz, administrative decision no 1/ŁB190/2021. Animals were randomly allocated to the treatment groups. The researcher who performed the measurements was unaware of the type of treatment applied to an animal.

### Human Donors

Human blood was collected from healthy donors under the guidelines of the Helsinki Declaration for human research and the study were approved by the Committee on the Ethics of Research in Human Experimentation at Medical University of Lodz (RNN/323/20/KE). Written informed consent, including detailed information regarding the study objectives, study design, risks, and benefits, was obtained from each individual before blood withdrawal. None of donors had taken aspirin or other drugs affecting platelet function for at least 10 days prior to blood collection or had a history suggestive of underlying haemostatic disorders. Blood was withdrawn to test tubes containing 0.105 mol/L sodium citrate (citrate: blood volume ratio 1:9) or hirudin with a special caution to avoid undesirable activation of circulating platelets. For the assessment of Src phosphorylation in blood platelets apyrase was added at final concentration of 0.02 U/mL.

### Flow Cytometry

Whole blood was incubated with activating factors for 5 minutes at room temperature (RT) and fixed with CellFix (BD Bioscience, Franklin Lakes, New Jersey, United States) at RT. The samples were then labelled with anti-CD41/PE antibodies, anti-JAM-A/FITC (BioLegend, San Diego, California, United States) or J10.4/FITC (Santa Cruz Biotechnology, Texas, United States) antibodies or IG _1_ κ/FITC (BioLegend, San Diego, California, United States) as an isotype control in final concentration of 15 µg/mL for 15 minutes at RT. Antibody titer was established on the basis of separate experiments. The lowest saturating concentration was chosen, i.e., when any higher concentrations did not increase the percentage of positive platelets compared with isotype antibodies. Prior to the measurements, the samples were diluted 1:30 with phosphate-buffered saline (PBS), and the assay was performed by recording 10,000 CD41/PE-positive events using a FACS Canto II flow cytometer (BD Bioscience, Franklin Lakes, New Jersey, United States). In the gated population of CD41/PE-positive events, the percentage fractions of the platelets positive with regard to JAM-A (above isotype cutoff) were measured.

### Adhesion to Fibrinogen and F11R/JAM-A under Flow Conditions

Blood platelet adhesion was assessed with the use of VenaFlux platform (Celix, Dublin, Ireland). Channels of Vena8 Endo+ biochip were coated with human fibrinogen (200 μg/mL) overnight at 4°C and blocked with 0.1% bovine serum albumin (BSA) for 1 hour at 4°C. Biochip was mounted on a thermo-controlled stage of an inverted AxioVert microscope (Carl Zeiss, Oberkochen, Germany), heating plate maintained the constant temperature of 37°C throughout the experiment. Prior to measurements, the channels were washed with saline. In the experiments involving co-coating of the chips with F11R/JAM-A, the chips were coated with 100 µg/mL Fbg and 100 µg/mL Fc-JAM-A. Blood samples were recalcified with CaCl _2_ in a concentration of 1 mM shortly before measurement. Whole blood was perfused through the channel at 40 dynes/cm ^2^ (∼890 s ^−1^ ) for 2 minutes. The channel was then perfused with the CellFix for 2 minutes at 5 dynes/cm ^2^ . Next, anti-CD41/PE antibodies were aspired to the channel, and incubated for 30 minutes. The channel was then washed with 100 µL of saline to remove any unbound antibodies. Images of labelled platelets were taken with AxioExaminer (Carl Zeiss, Oberkochen, Germany) microscope using a 20× objective focusing on at least five different region of interest (ROI). Area covered by platelets was quantified with the use of Fiji (ImageJ) preceded by segmentation step performed with the use of Ilastik software. 20 Additional experiments were performed to quantify the number of platelets adhering as a function of time. Perfusion conditions were as described above. Adhesion events in real time were recorded. Movie sequences were analysed with the use of TrackMate plugin for ImageJ.

### Adhesion to Fibrin and F11R/JAM-A under Static Conditions

For the static adhesion study, the surface of Ibidi µ-Slide 15 wells (Ibidi, Martinsried, Germany) was coated overnight with fibrinogen (37.5 μg/mL) at 4°C. After coating, the excess of coating solution was removed, so the reaction with calcified (1 mM) thrombin (1 U/mL over 15 minutes, 37°C) formed a flat net instead of a fully three-dimensional mesh, which was critical to achieving a uniform plane of focus for platelet observation. Surfaces were additionally treated with recombinant Fc-F11R /JAM-A (100 µg/mL) or BSA (0.1%). Platelet-rich plasma was recalcified and incubated over the surfaces for 30 minutes. Next, the wells were washed with Tyrode solution and left to incubate for an additional 30 minutes. Adhered platelets were fixed using CellFix solution, permeabilized with Triton X-100 (0.1%), and stained with Phalloidin for 30 minutes at RT, and excess staining was removed by washing with 100 µL of PBS.

Images were taken in five different fields of view in each well with the use of AxioExaminer microscope (Carl Zeiss, Oberkochen, Germany). Ilastik software was used for machine learning-based classification and quantification of distinct morphological types of blood platelets. To this end a modified protocol described previously by Pike et al was used. 21 Briefly, a model was trained to differentiate three morphological types of platelets: (1) nonactivated, i.e., round-shaped, devoid of filopodia; (2) presenting at least one filopodium but devoid of lamellipodia; and (3) presenting lamellipodium or fully spread. Images were analysed with the trained model to obtain percentage of platelets presenting each of the phenotypes. Trained Ilastik model is available in ZENODO repository (https://doi.org/10.5281/zenodo.13378736).

### Confocal Microscopy

Blood platelets incorporated into thrombi in VenaFlux chips channels were stained with anti-CD41/PE and anti-JAM-A/AlexaFluor488 antibodies as described above. Blood platelets adhered to fibrinogen and F11R/JAM-A in VenaFlux chips channels were stained with anti-CD41/PE as described above. F11R/JAM-A staining pattern was visualised using a confocal microscope (Nikon D-Eclipse C1) and analysed with EZ-C1 version 3.6 software (Nikon, Japan).

### Fab Enzymatic Preparation

For the preparation of Fabs we used the ficin enzymatic cleaving capacity provided by Pierce murine IgG1 Fab Micro Preparation Kit (ThermoFisher Scientific), in accordance with manufacturers Fab preparation protocol. Successful fragmentation of J10.4 to Fabs of J10.4 was confirmed with the use of SDS-PAGE.

### Whole-Blood Impedance Aggregometry

The measurements were performed using Multiplate analyzer (Hoffmann-La Roche, Basel, Switzerland) according to the manufacturer's instructions. Briefly, whole blood was preincubated with full J10.4 antibodies or their Fabs for 5 minutes at RT, then 300 µL of blood was transferred into the measurement cell and diluted with 300 μL 0.9% NaCl and preheated to 37°C for another 3 minutes. In selected experiments 0.5 μM ADP (final concentration) was added and platelet aggregation was recorded continuously for 10 minutes. Area under the curve (AUC) was monitored. All measurements were completed within 3 hours of blood collection.

### Thrombus Formation Analysis

Total Thrombus formation Analysis System (Zacros, Fujimori Kogyo Co. Ltd., Tokyo, Japan) was used to analyze in vitro thrombus formation. 22 23 In this method the blood is perfused through a capillary coated with a specific substrate. The perfusion pressure that increases as the thrombi buildup is a measure of the capillary occlusion. Reaching of a preset pressure value is considered as a total occlusion of the capillary. The time to the total occlusion as well as AUC of pressure changes over time are the quantitative parameters assessed in the method. Depending on the substrate coating the capillary and blood preparation, the assay measures primary or total haemostasis. In the first case, hirudin-anticoagulated blood is perfused through collagen-coated capillary, whereas in the second case citrated and recalcified blood is perfused through collagen and thromboplastin-coated capillary. Blocking agents (IgG, Fabs, and soluble F11R/JAM-A His-tagged protein) were added to the whole blood 5 minutes prior to perfusion.

### Src Phosphorylation Analysis

Platelet-rich plasma was obtained from whole blood by centrifugation at 200 g for 12 minutes and carefully collected to avoid contamination with red blood cells. Platelets were pelleted by centrifugation at 700 g for 15 minutes and suspended in Tyrode's buffer (134 mM NaCl, 12 mM NaHCO _3_ , 2.9 mM KCl, 0.34 mM Na _2_ HPO _4_ , 1 mM MgCl _2_ , 10 mM HEPES, 5 mM glucose, pH 7.4). Aliquots of platelet suspension (150 × 10 ^3^ /µL) were added to wells of a 6-well plate coated with 100 µg/mL fibrinogen. Then, Fab fragments of J10.4 or isotype antibodies were added to final concentration of 50 µg/mL. After 1-hour incubation at 37°C nonadherent platelets were removed. Adherent platelets were lysed by incubation with lysis buffer (1% NP40, 0.2% sodium deoxycholate, 150 mM NaCl, 50 mM Tris pH 7.5, 1 mM PMSF, protease and phosphatase inhibitors cocktail) for 30 minutes at 4°C. Lysates were centrifuged at 16,000 g for 20 minutes at 4°C. The supernatants were stored at −80°C. To evaluate the level of Src phosphorylation samples with equal total protein content were loaded and separated on SDS–PAGE gels (Mini-PROTEAN TGX Gels; Bio-Rad Laboratories, Hercules, California, United States) and then transferred onto polyvinylidene fluoride membranes (Bio-Rad Laboratories). For the analysis of Src levels primary antibodies: goat anti-Src (R&D Systems) and rabbit anti-phospho-Src (Y416) (R&D Systems) were used. GAPDH was used as a loading control and detected using rabbit anti-GAPDH (Abcam, Cambridge, GB). Respective horseradish peroxidase-conjugated anti-goat or anti-rabbit secondary antibodies (ThermoFisher Scientific) were used. The signal was detected by measuring the chemiluminescence with Pierce ECL Western Blotting Substrate (ThermoFisher Scientific). Bands' intensity was quantified with the use of ImageJ. The intensities of the phospho-Src bands were normalized to the respective Src and GAPDH bands.

### Ferric Chloride-Induced Injury

Total anaesthesia was induced using mix of ketamine (100 mg/kg) and xylazine (10 mg/kg) protocol. The mixture was injected intraperitoneally. Intravenous administration of staining antibodies and tested substances was performed by injection to the retro-orbital plexus after confirming full anaesthesia. All surgical procedures were performed using dissecting microscope to aid the operator. Carotid artery was exposed as described in detail in Supplementary Material S1 (available in the online version only). A strip of a black polyethylene wrap and a strip of filter paper (Whatman, Sanford, Maine, United States) were threaded under the blood vessel to provide isolated field of view and a vehicle for FeCl _3_ . The BV11 antibodies were administered intravenous into the retro-orbital plexus. To saturate the filter paper, 3 µL of the 10% ferric chloride was applied. Immediately after administration of FeCl _3_ , the probe of laser Doppler flowmeter was placed directly over the cranial end of the carotid at a height of approximately 1 mm and data collection ensued. Time of the experiment was measured starting with the application of FeCl _3_ solution to the paper strip. The measurements were taken for 30 minutes or until reduction of the blood flow to below 10% of the initial value expressed in arbitrary units (LDU) for more than 2 minutes.

Data were collected with the use of a ML191 Blood Flowmeter (ADInstruments, Colorado Springs, Colorado, United States). Doppler shift was analysed and recalculated to LDF output signal by the flowmeter and transferred to ML870 PowerLab 8/30 data acquisition system. All the LDF data were recorded as a function of time by Chart 5 software.

### Statistical Analysis

Data were presented as mean ± standard deviation or median and interquartile range (IQR), depending on the normality of data distribution. The Shapiro–Wilk test and Levene's test were used to confirm that the data were normally distributed and homogenous. For normally distributed and homoscedastic variables, the statistical significance of differences between two groups was estimated using the paired or the unpaired Student's *t* -test; for variables that departed from normality and/or variance homogeneity, Mann–Whitney U test was applied instead. To compare differences between more than two groups with a control group, analysis of variance for repeated measures and the Dunnett's post hoc test for multiple comparisons were used. In time-dependent analyses, the Kaplan–Meier model was adopted. Comparison of time to occlusion occurrence was performed with the use of log-rank (Mantel–Cox) test. To compare the fractions of samples analysed with the use of T-TAS in which embolisation occurs and to compare the fractions of animals, which developed occlusion, the Fisher's exact test was used. Statistica v. 13.1 (Dell Inc., Tulsa, Oklahoma, United States), GraphPad Prism v.9 (San Diego, California, United States) were used for statistical calculations and to draw charts.

## Results

### Contribution of Homophilic F11R/JAM-A Interactions to Platelet Adhesion under Flow Conditions

**Fig. 1:**
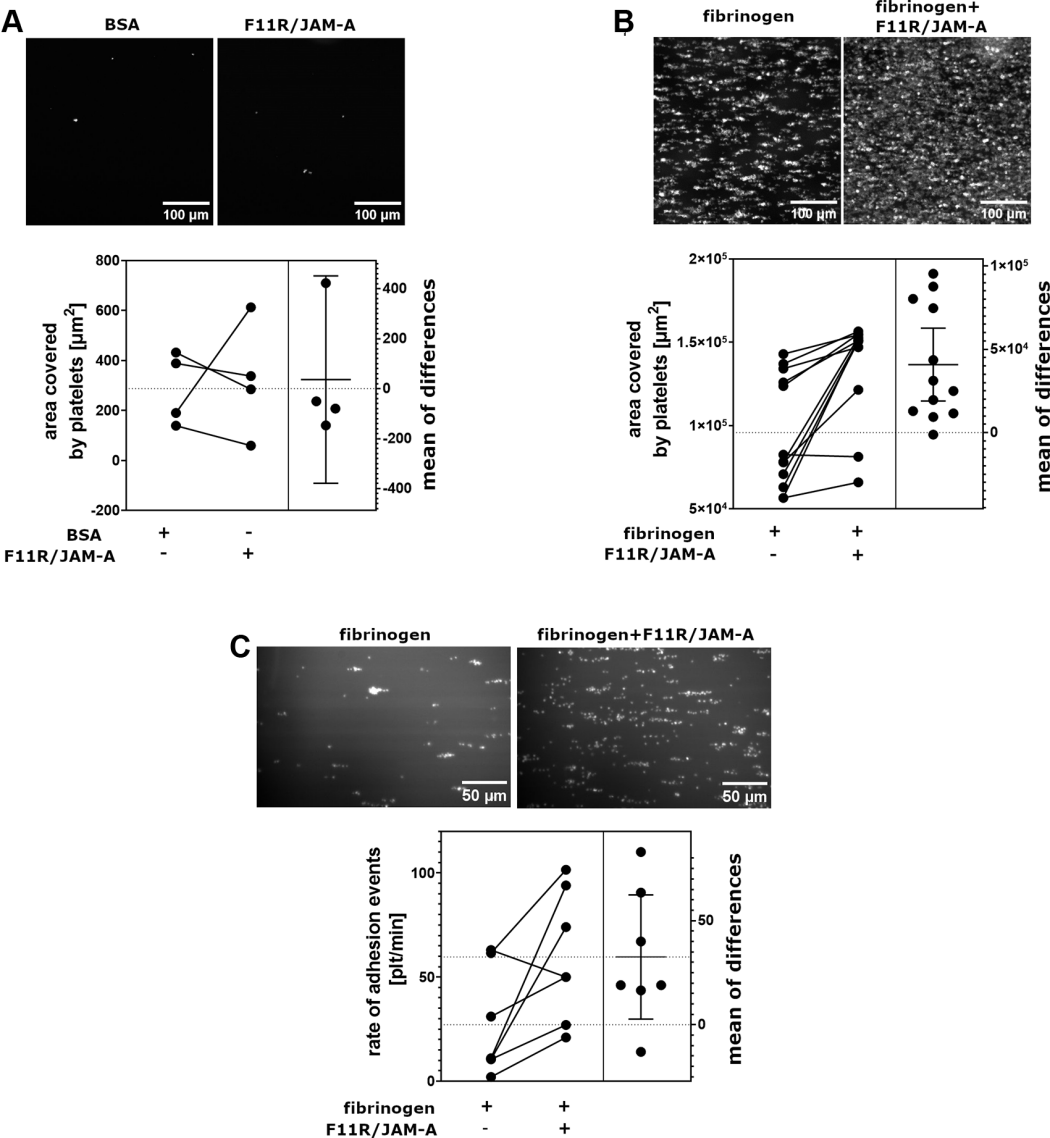
Capability of surface-bound F11R/JAM-A to capture blood platelets under flow conditions. ( **A** ) Platelets firmly adhered to bovine serum albumin (BSA)-coated or Fc-F11R/JAM-A-coated surface under flow conditions at 40 dyne/cm ^2^ (∼890 s ^−1^ ); ( **B** ) platelets firmly adhered to fibrinogen-coated surface or fibrinogen and Fc-F11R/JAM-A-coated surface under flow conditions at 40 dyne/cm ^2^ (∼890 s ^−1^ ) *P* = 0.0017, paired two-tailed *t* -test, *n =* 12. ( **C** ) Number of firmly adhered platelets to a surface coated with fibrinogen and Fc-F11R/JAM-A was monitored in real-time at 40 dyne/cm ^2^ (∼890 s ^−1^ ); *P* = 0.037, paired two-tailed Student's *t* -test, *n* = 7. Each pair of values represents a sample from individual donor. Right panel of each chart presents differences calculated for each pair of values shown on the left panel (coverage on Fc-F11R/JAM-A–coated surface upon subtraction of the fibrinogen or BSA alone–coated surface) shown as mean ± standard deviation.

Surface coated with F11R/JAM-A alone had no capability to capture and immobilise blood platelets under flow conditions ( Fig. 1A ). This lack of adhesion was also observed at lower, venous shear force conditions ( Supplementary Fig. S1 , available in the online version only). However, when the surface was coated with the mix of both F11R/JAM-A and fibrinogen more platelets adhered to such combination of proteins than to fibrinogen alone ( Fig. 1B ). Since the increased coverage observed in the presence of F11R/JAM-A could be assigned either to an increased number of platelets adhering in a period of time, or to an increased spreading of adhered platelets, in order to dispel these doubts the additional experiments were performed where adhesion events were monitored in real time. Quantitative analyses revealed increased frequency of adhesion events on surface covered with fibrinogen in a combination with F11R/JAM-A when compared with adhesion on fibrinogen alone ( Fig. 1C ), confirming that the presence of F11R/JAM-A on the surface allowed more efficient capture of flowing platelets.

### Compounds Blocking F11R/JAM-A-dependent Interactions

As a next step we aimed at verifying whether F11R/JAM-A-dependent enhancement of platelets interactions with fibrinogen described above could contribute to thrombus formation. To this end, we used two approaches to block F11R/JAM-A homophilic interactions. One of them being J10.4 monoclonal antibodies with a defined ability to block homophilic interactions of F11R/JAM-A 24 25 and the other was the use of soluble form of F11R/JAM-A.

Taking into consideration that monoclonal F11 antibodies against F11R/JAM-A were previously reported to activate blood platelets in FcγRII-dependent manner, we first tested J10.4 clone to exclude its ability to exert similar effect. Interestingly, a whole-blood aggregometry assay revealed that J10.4 antibodies induced platelet aggregation and caused potentiation effect of subthreshold ADP concentration ( Supplementary Fig. S2 , available in the online version only). Therefore, in order to use these antibodies as a blocking factor devoid of activating effect, their Fab fragments were prepared as described in the methods section ( Supplementary Fig. S3 , available in the online version only). Prepared fragments were tested to assure the lack of the stimulatory effect. As expected Fab fragments of J10.4 did not increase ADP-induced platelet activation ( Supplementary Fig. S2 , available in the online version only). Murine isotype Fab fragments were prepared according to the same protocol to be used as control.

### Contribution of Homophilic F11R/JAM-A Interactions to Thrombus Formation Assessed in Total Thrombus Formation Analysis System

**Fig. 2:**
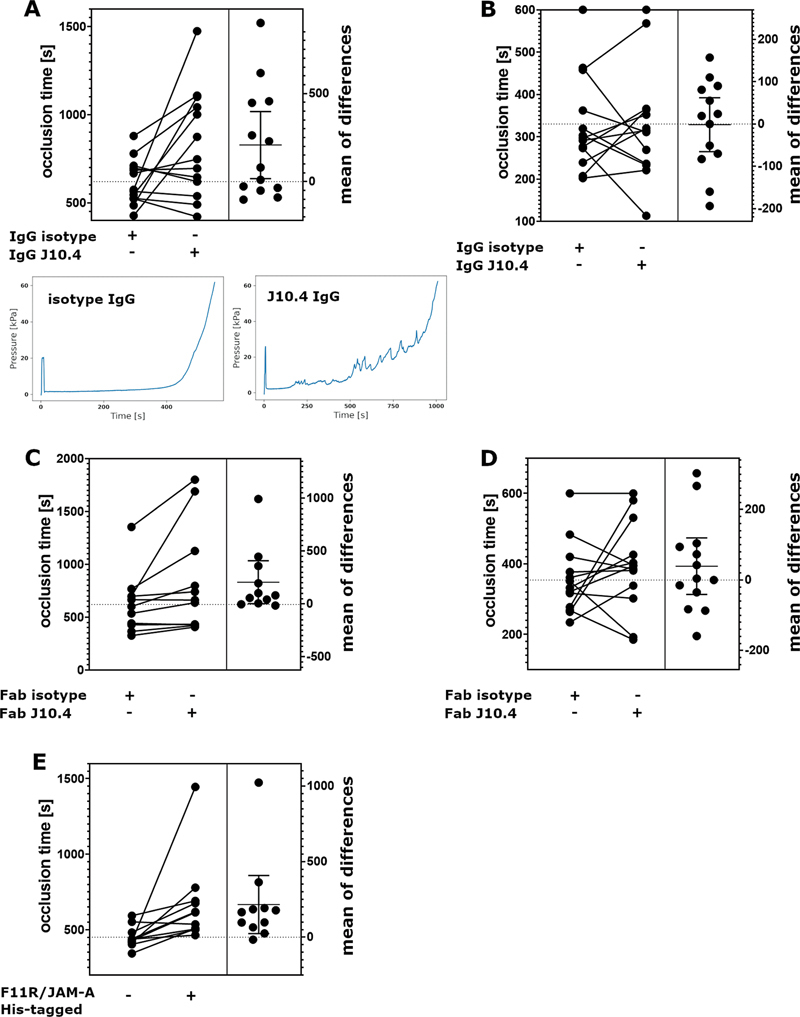
Effects of J10.4 antibodies and soluble F11R/JAM-A on thrombus formation. Effects of J10.4 antibodies on: ( **A** ) time of occlusive thrombus formation, *P* = 0.035 two-tailed paired Student's *t* -test ( *n* = 13), panels below show representative registrations of occlusive thrombus formation and the occurrence of embolisation in the presence of J10.4 antibodies in comparison to unaffected thrombosis in the presence of isotype IgG; ( **B** ) primary haemostasis, not significant (n.s.) Effects of Fab fragments of J10.4 antibodies on: ( **C** ) time of occlusive thrombus formation, *P* = 0.0068 Wilcoxon matched-pairs signed rank test ( *n* = 11); ( **D** ) primary haemostasis, n.s.; ( **E** ) effects of soluble His-tagged F11R/JAM-A on time of occlusive thrombus formation, *P* = 0.002 Wilcoxon matched-pairs signed rank test ( *n* = 11); each pair of values represents a sample from individual donor. Right panels of the charts present differences calculated for each pair of values shown on the left panels (the aggregation in the presence of J10.4 or soluble F11R/JAM-A upon subtraction of the aggregation in the presence of isotype or vehicle) shown as mean ± standard deviation.

In most of the blood samples treated with J10.4 antibodies the time to formation of an occlusive thrombus was significantly increased when compared with isotype IgG treated samples ( Fig. 2A ) indicating that antibodies impaired thrombus formation. In some donors however, as depicted on the figure, the effect was opposite. Additionally, in 7 out of 13 samples treated with J10.4 antibodies, the process of thrombus growth was interrupted with drops of pressure reflecting thrombi embolization, which was not observed in the control group (sample tracings shown under Fig. 2A ). This fraction of embolisation occurrence was statistically significant ( *p* = 0.0052, Fisher's exact test). In none of the donors, however, thrombus formation was entirely abrogated. Importantly, no effect of J10.4 antibodies was observed when primary haemostasis capacity was evaluated ( Fig. 2B ).

Similarly to the full J10.4 antibodies, Fab fragments of J10.4 significantly delayed time to occlusion in the total haemostasis assay ( Fig. 2C ) and in contrast to the full antibodies in none of the donors the opposite effect was observed. Interestingly, in one donor out of 11, the thrombus formation was completely inhibited. Primary haemostasis was not affected by Fab fragments of J10.4 ( Fig. 2D ).

To further confirm the effect of the blockade of homophilic F11R/JAM-A interactions on total thrombus formation, soluble recombinant F11R/JAM-A molecule with His tag was used. Its presence significantly delayed thrombus formation ( Fig. 2E ).

### Western Blot

To verify whether the inhibition of F11R/JAM-A cis-dimerisation using J10.4 Fab fragments affects platelet intracellular signalling, we assessed Y416 phosphorylation of Src in blood platelets adhering to fibrinogen in the presence of either J10.4 Fab or isotype Fab. No significant effect of J10.4 Fab on the level of phospho-Src was observed ( Supplementary Fig. S4 , available in the online version only).

### Flow Cytometry Analysis

**Fig. 3:**
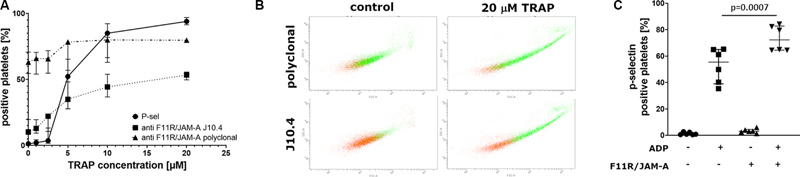
Flow cytometry analysis of F11R/JAM-A expression and platelet activation (P-selectin exposure). Effects of TRAP on total or monomeric F11R/JAM-A expression: ( **A** ) percentage of blood platelets positive for monomeric F11R/JAM-A (J10.4 antibody staining), total F11R/JAM-A (polyclonal antibody staining) in comparison with percentage of platelet activation level revealed by P-selectin staining ( *n* = 3–7); data presented as median with interquartile range [IQR] ( **B** ) representative dot-plots showing location of blood platelets positively stained with polyclonal or J10.4 antibodies (green dots) in the population of CD41-gated blood platelets (red dots) either resting or activated with 20 µM TRAP; ( **C** ) effects of soluble His-tagged F11R/JAM-A alone and in combination with ADP on platelet activation, one-way analysis of variance ( *P* < 0.0001) followed by Tukey's post hoc multiple comparisons test ( *P* -value adjusted for multiple comparisons depicted on the plot), *n* = 6; data presented as median with IQR.

Flow cytometry was used to evaluate the binding of monoclonal J10.4 antibodies to resting and activated blood platelets. The level of platelet activation was assessed with the monitoring of the P-selectin exposure. As shown on Fig. 3A only approx. 10% of resting platelets was positively stained with J10.4 antibodies. This J10.4-positive fraction increased gradually along with platelets stimulation by TRAP (1–20 µM), but it reached only 50% compared with nearly 100% of platelet P-selectin exposure under the same conditions. Interestingly, platelets binding J10.4 antibodies were located mainly in the fraction of larger platelets and platelet aggregates ( Fig 3B ).

In turn, when polyclonal antibodies against F11R/JAM-A were used, approximately 60% of resting platelets were positive and this number only slightly increased with platelet activation ( Fig 3A ).

Flow cytometry was also used to verify the effects of soluble His-tagged F11R/JAM-A on platelet reactivity. As shown on Fig. 3C , His-tagged F11R/JAM-A enhanced platelet response to ADP (as revealed by an increased P-selectin exposure) when used in the same concentration, which delayed thrombus formation in T-TAS analyses. .

### Expression of F11R/JAM-A on Activated and Adhered Blood Platelets

**Fig. 4:**
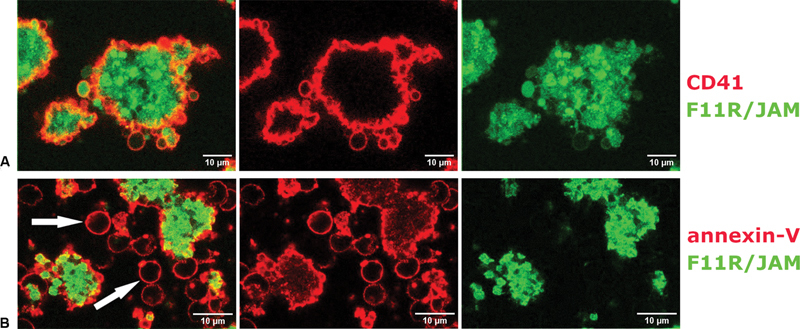
Expression of F11R/JAM-A on blood platelets in thrombus formed on collagen. Images show a confocal section in the medial position of the thrombus (100× oil objective). Red staining represents ( **A** ) anti-CD-41 labeling or ( **B** ) annexin V labeling and green staining represents anti-F11R/JAM-A labeling. Arrows point at ballooning platelets. Representative examples of images acquired on blood samples from three different donors.

As flow cytometry analysis showed that the expression of F11R/JAM-A increases upon platelet stimulation with the agonist, confocal imaging was performed to evaluate if F11R/JAM-A-positive staining changes throughout thrombi. We revealed no specific gradation of fluorescence intensity between the core and the outer layers of thrombus, suggesting that platelets do not differ in terms of F11R/JAM-A expression throughout the body of thrombus ( Fig. 4A, B ). Since staining was performed without permeabilisation it could be assumed that only the pool of the protein presented on platelet surface is visible. Interestingly, structures that may be identified as procoagulant platelets (the annexin V positive, balloon-shaped objects located on the borders or outside the body of thrombus) were characterised by relatively low expression of F11R/JAM-A when compared with platelets located in the body of thrombus ( Fig. 4B ).

### The Effects of F11R/JAM-A on Platelet Adhesion to Fibrin

**Fig. 5:**
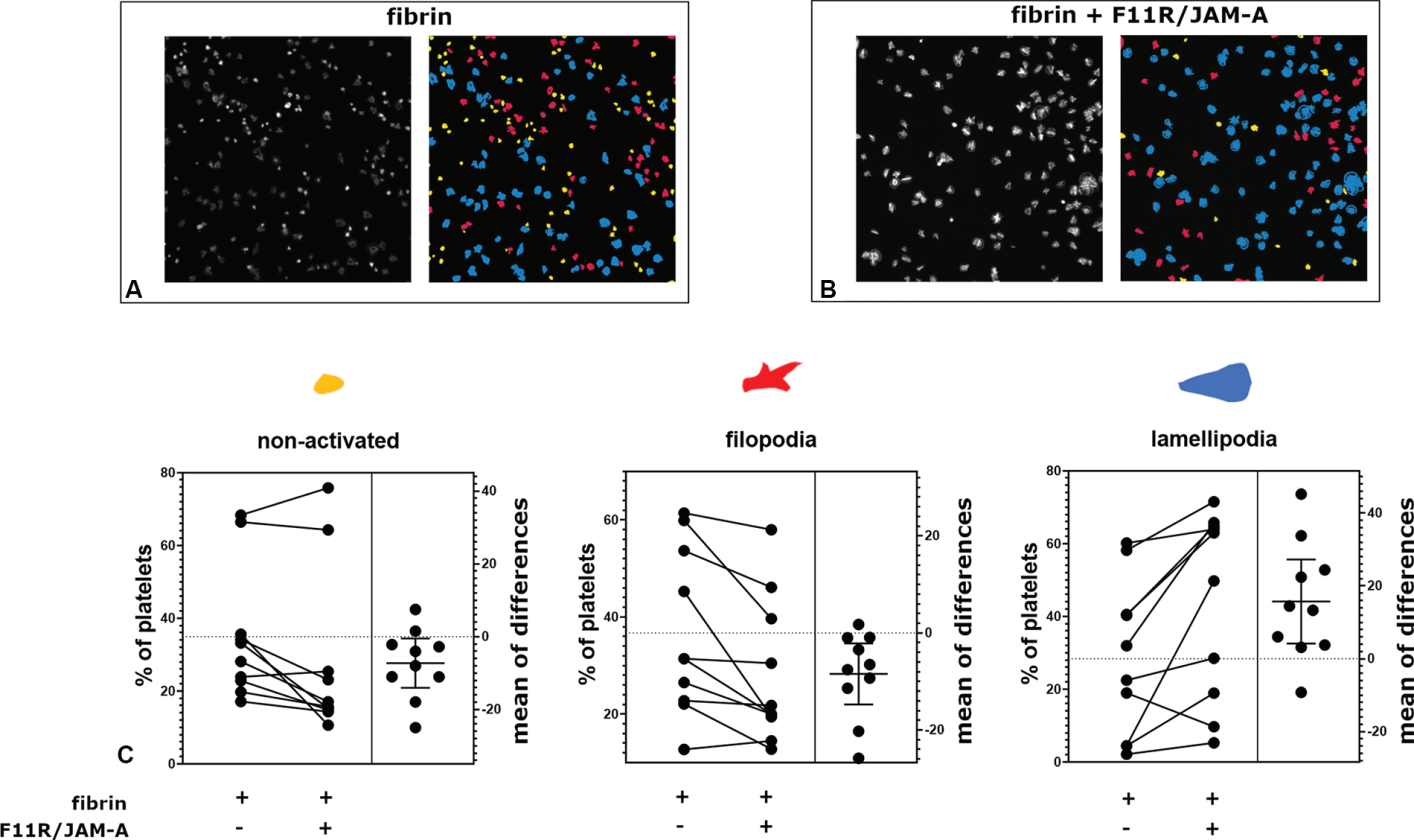
Effects of F11R/JAM-A on morphology of blood platelets adhered to fibrin ( **A** ) representative image of platelets adhered to fibrin (left panel) with platelets assigned to one of three morphological classes by the trained classifier (right panel); ( **B** ) representative image of platelets adhered to fibrin and Fc-F11R/JAM-A (left panel) with platelets assigned to one of three morphological classes by the trained classifier (right panel). ( **C** ) Comparison of fractions of platelets assigned to one of three morphological classes: nonactivated *P* < 0.05, filopodia *P* < 0.05, lamellipodia *P* < 0.05 (paired *t* -test), *n* = 10. Each pair of values represents a sample from individual donor. Right panels of the charts present differences calculated for each pair of values shown on the left panels (adhesion to fibrin and Fc-F11R/JAM-A upon the subtraction of adhesion to fibrin alone) shown as mean ± standard deviation.

Previous experiments showed that platelet F11R/JAM-A plays a role in thrombus formation, but it is not related to platelet aggregation. Therefore, we tested how F11R/JAM-A presence on the surface can affect platelet adhesion to fibrin-like structure. As shown on Fig. 5 , platelets adhering to fibrin-like structure in the presence of F11R/JAM-A more often acquired lamellipodial morphology than platelets adhering to fibrin-like mesh alone. This was accompanied by a significant decrease of filopodial platelets.

### Contribution of Homophilic F11R/JAM-A Interactions to Thrombus Formation In Vivo

The effects of F11R/JAM-A blockade were tested in an in vivo thrombosis models in carotid artery in mouse. As the blocking agent, anti F11R/JAM-A antibodies (clone BV11) were used that were previously shown to inhibit homophilic interactions of F11R/JAM-A 6 and to reduce migration of neutrophils through venules in inflammatory conditions in mice. 26 Isotype IgGs were used as a control.

**Fig. 6:**
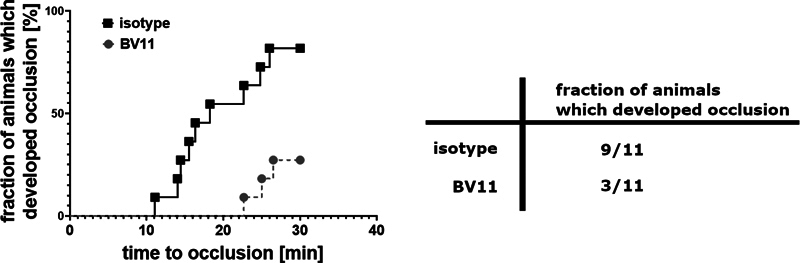
Effects of BV11 antibodies on thrombus formation in a model of experimental thrombosis in mouse carotid artery. Comparison of time to occlusion occurrence measured by means of laser Doppler flowmetry; *P* = 0.0026, log-rank (Mantel–Cox) test, *n* = 11; the comparison of proportions of animals that developed occlusion; *P* = 0.03, Fisher's exact test, *n* = 11.

A rate of occlusion, measured as a decrease in blood flow, was significantly lower in BV11-treated animals than in isotype-treated control. Also, a lower number of animals developed full occlusion during observation period in BV11-treated animals than in isotype-treated group ( Fig. 6 ). To rule out that lower incidence of occlusion was caused by platelet depleting effect of administering BV11 antibodies, in a separate group of animals an effect of BV11 antibodies on platelet count was tested. In the animals treated with BV11 platelet count was decreased from 947 (831; 970) × 10 ^3^ plt/µL (median with IQR) to 524 (492; 1,038) in a period of 30 minutes from the time of antibody injection.

## Discussion

### Contribution of F11R/JAM-A to Platelet Immobilisation under Flow Conditions

Homophilic interactions between F11R/JAM-A expressed on blood platelets and that immobilised on a surface were proven to support platelet adhesion and spreading under static conditions. 11 16 Our earlier research showed that in vivo under flow conditions F11R/JAM-A-dependent interactions were involved in transient adhesion of platelets to inflamed endothelium. 19 In the present work we aimed at elucidating whether homophilic interactions of F11R/JAM-A, which apparently facilitate platelet adhesion in static conditions, would provide such support under flow conditions and how these interactions translate to thrombus formation.

According to our present study, interactions of flowing platelets with F11R/JAM-A immobilised on a surface as the sole adhesion protein were not sufficient to capture and immobilise platelets, even under low-shear, venous flow conditions. On the other hand, when F11R/JAM-A was co-coated with fibrinogen, it turned out to play a marked supporting role in recruiting platelets to the surface under high shear arterial flow conditions. Therefore, although F11R/JAM-A homophilic interactions per se are not sufficient to immobilise a flowing platelet, they may increase the efficiency of adhesion to surfaces where other proadhesive proteins are present. Such a milieu is present in thrombus where activated platelets presenting a plethora of proadhesive molecules are interweaved with fibrin. Whether this F11R/JAM-A-mediated facilitation of adhesion is due to a physical tethering of flowing platelet by homophilic interaction between surface-bound F11R/JAM-A and that present on platelet or this interaction only primes platelet to develop physical interaction via another adhesive proteins remains to be elucidated. However, taking into account that even at low shear forces F11R/JAM-A-mediated interactions alone were not sufficient to arrest the flowing platelets suggests the latter explanation more likely.

It is not clear why homophilic interactions of F11R/JAM-A alone can mediate platelet adhesion and activation under static conditions, but they are insufficient on their own under the flow conditions. It may be assumed that in flow these interactions are too short lasting or that downstream signal is too weak to activate platelet to an extent required for immobilisation. Under static conditions in turn, the period of interactions could be sufficient to trigger the downstream signal resulting in platelet adhesion.

### Contribution of F11R/JAM-A to Thrombus Formation

To understand whether this F11R/JAM-A-mediated facilitation of adhesion translates to thrombus formation we looked at the effects of compounds blocking F11R/JAM-A homophilic interactions on formation of occlusive thrombus.

In the first place we used monoclonal J10.4 antibodies that are very well defined in terms of binding site on F11R/JAM-A molecule, and which were shown to bind specifically to monomeric F11R/JAM-A and to inhibit its homophilic cis-dimerisation. 25 The J10.4 antibodies themselves, however, activated blood platelets in Fc fragment-dependent fashion that was similar to previously published studies, which had shown that stimulatory monoclonal F11 antibodies alone activated platelets in an FcγRIIa-dependent manner. 10 Therefore, we performed these experiments in parallel with their Fab fragments devoid of the activating effect. Application of either full antibodies or their Fab fragments resulted in significant prolongation of time to formation of occlusive thrombus. The effect of J10.4 full antibodies, however, was not unequivocal. In some of donors it caused an acceleration of occlusive thrombosis rather than a delay. Moreover, in a significant number of donors the antibodies application resulted in disturbed formation of thrombus characterised by repetitive embolisation. This may be explained by the fact that activating effect of full antibodies competed with their inhibitory effect.

The use of a soluble form of F11R/JAM-A was another mean of blocking F11R/JAM-A–dependent interactions. His tag F11R/JAM-A delayed the formation of occlusive thrombus. Two possible mechanisms can explain this effect. Soluble His tag F11R/JAM-A, as a monomeric protein, can bind to monomeric F11R/JAM-A on blood platelet in the site responsible for homodimeric cis-dimerisation, thus preventing cis-homodimerisation of platelet portion of this protein. His tag F11R/JAM-A would therefore act in a way similar to J10.4 antibodies. However, this hypothesis is not supported by the fact that under physiological pH F11R/JAM-A tends to form cis-dimers; 6 therefore, we rather opt that the His tag F11R/JAM-A is more likely to be present as a dimer in an experimental environment. As such it presumably binds to platelet F11R/JAM-A in trans-configuration blocking the interplatelet interactions between F11R/JAM-A molecules located on flowing platelets and platelets already incorporated in thrombus.

Our finding that soluble F11R/JAM-A delayed formation of occlusive thrombus is in contrast to the work of Rath et al who showed that soluble F11R/JAM-A enhanced thrombus formation in flow chamber assay and increased platelet reactivity. 18 The soluble form used in the cited studies was, as the authors assumed, dimeric under the experimental conditions. To evaluate whether soluble form used in our studies exerts similar effects on platelet activation as that described by Rath et al we performed similar assays and found that indeed it increased blood platelets response to ADP. Why a molecule that induced activation of blood platelets could cause a delay in thrombus formation in another experimental conditions? To our understanding, this apparent disagreement may be due to the dual nature of the effects mediated by soluble F11R/JAM-A. Binding of soluble form of the protein to platelet F11R/JAM-A has two consequences: one of them being the sending of a downstream signal for platelet activation as shown by Rath et al, and the other being the blocking the interaction of platelet F11R/JAM-A molecule with its counterpart on another blood platelet. While the former effect is obviously prothrombotic, the latter is antithrombotic, as shown by our experiments with J10.4 antibodies that block cis-dimerisation.

Importantly, neither of the compounds caused a definitive abrogation of the formation of occlusive thrombi. None of them also compromised primary haemostasis. Therefore, we conclude that although homophilic interactions of F11R/JAM-A between platelets facilitate formation of thrombus they are not a decisive factor.

In accordance with the in vitro results, the blockade of F11R/JAM-A interactions in an animal model of thrombosis caused a significant decrease of thrombus formation. However, in this case, antibodies were found to decrease the number of circulating platelets. Although the final level of circulating platelets was in the range that, according to literature, 27 28 still ensures haemostasis in mice, the observed outcome of reduced thrombosis could be partially attributed to this effect.

The question arises as to which of the type of interactions of F11R/JAM-A, cis- or trans- are affected by blocking antibodies and soluble F11R/JAM-A. Monoclonal J10.4 antibodies were shown to bind solely to F11R/JAM-A monomers and to inhibit cis-dimerisation. 25 According to some studies cis-dimerisation in turn is necessary for trans-dimerisation. 8 The antibodies would then inhibit trans-homodimerisation indirectly by inhibiting cis-dimerisation. Soluble His tag F11R/JAM-A is expressed as a monomeric protein but according to Bazzoni et al in physiological pH the protein forms cis-homodimers, which renders cis-homodimerisation sites inaccessible. 6 Since cis- and trans-homodimerisation occurs at different sites 8 soluble His tag F11R/JAM-A still could bind to platelet F11R/JAM-A via trans-homodimerisation site. At the same time cis-dimerisation of F11R/JAM-A per se does not enhance intracellular signalling via Src kinase during platelet adhesion, as shown by the lack of the effect on its phosphorylation in platelets adhering to fibrinogen in the presence of J10.4 Fabs. This way of reasoning therefore advocates the notion that the type of F11R/JAM-A interactions involved in thrombus formation are the interactions between two molecules of F11R/JAM-A located on opposing cells and that the dimerisation of monomeric F11R/JAM-A facilitates this process.

This trans-homodimerisation of platelet F11R/JAM-A may occur not only with F11R/JAM-A presented on another platelets in thrombus, but also with soluble F11R/JAM-A entrapped in growing thrombus either as soluble F11R/JAM-A molecules or as F11R/JAM-A rich microvesicles. As shown by Rath et al 18 soluble F11R/JAM-A increases platelet reactivity but, by their own admittance, humoral levels of soluble JAM-A required to achieve this effect in vitro are several-fold higher than those observed naturally in serum. As they suggested, it could be assumed that concentration of soluble F11R/JAM-A is increased locally in thrombus milieu due to accumulation of the protein. Considering the fact that activated platelets can produce F11R/JAM-A-rich microvesicles, 29 this soluble fraction of the protein may actually become stationary from the functional point of view by the way of incorporating to growing thrombus. Therefore, we suggest that this localised high concentration of F11R/JAM-A may be present in stationary phase either as accumulated soluble F11R/JAM-A or as F11R/JAM-A presented by platelets, which were already incorporated in thrombus and thus provide target-rich environment for local interactions.

In this context, the abundance of F11R/JAM-A accessible for trans-homophilic interactions on the surface of resting and activated blood platelets is an important factor. As shown by the staining with polyclonal antibodies, F11R/JAM-A is present on a majority of both resting and activated platelets, which is consistent with previously published reports. 13 However, only approximately 10% of resting platelets bound J10.4, the antibodies that are known to bind specifically to monomeric F11R/JAM, 25 while according to previous reports, monomeric F11R/JAM-A dominates in resting platelets, where it is associated with αIIbβ _3_ . 14 It is therefore expected that majority of resting platelets should bind J10.4 antibodies. The inconsistency could be possibly explained by low accessibility of the epitope on F11R/JAM-A, which binds J10.4 antibodies under conditions when the protein is in complex with αIIbβ _3_ . When F11R/JAM-A dissociates from αIIbβ _3_ during platelet activation, the accessibility of F11R/JAM-A to J10.4 increases _._ Since monomeric F11R/JAM-A dissociated from αIIbβ _3_ plausibly tends to form dimers, 13 the capacity of J10.4 binding is limited. It would explain why only up to approximately 50% of activated platelets became positive against J10.4 in the same conditions when more than 80% was stained with polyclonal antibodies.

It was reported earlier that the interaction of platelet F11R/JAM-A with JAM-A enriched fibrinogen coating in static conditions changes platelet shape to more flattened when compared with fibrinogen alone, 18 which shows that homophilic interactions of F11R/JAM-A have yet another intriguing consequence, such as the modulation of platelet phenotype. Additionally, recent studies showed platelets to acquire different phenotypes depending on whether they interact with fibrin or with fibrinogen. 30 While platelets interacting with fibrin formed protrusions and remained stationary, these adhered to fibrinogen were flattened and acquired migratory phenotype. Since fibrin is a component of thrombus, we wondered whether homophilic interactions of F11R/JAM-A could play a role in this process. Our experiments showed that a shift in platelet shape occurred in platelets adhering to fibrin in the presence of F11R/JAM-A. It suggests that F11R/JAM-A not only facilitates immobilisation of flowing platelets, but also plays a role in the process of thrombus organisation. Interestingly, as shown by confocal imaging of F11R/JAM-A staining in thrombus, the protein was present in platelets across thrombus with relatively less to none staining on platelets, which could be identified as procoagulant platelets, i.e., ballooned-shaped annexin V positive ones. 31 It supports a notion that the regulatory role of F11R/JAM-A is important in the inner part of thrombus rather than in its outer layers. Besides F11R/JAM-A contribution to platelet–platelet interactions, the protein can also take part in platelet–endothelium interactions where platelets take part in sealing gaps between adjacent endothelial cells in inflamed vascular wall, a process that has been recently described. 30

## Conclusion

We show that interactions of F11R/JAM-A located on flowing platelets with its surface-bound counterpart enhance platelets binding to fibrinogen under high shear stress conditions. These homophilic interactions modulate thrombus formation but are not indispensable for its completion. F11R/JAM-A plays a role in acquiring by platelets of adhesive phenotype during interaction with fibrin mesh. While previously published studies pointed at a significant role of soluble F11R/JAM-A in priming platelets during thrombus formation, our results address the role of surface-bound F11R/JAM-A in this process.

## Notes

**What is known about this topic?**

- F11R/JAM-A mediates blood platelets' adhesion under static conditions.
- Soluble F11R/JAM-A activates blood platelets.

**What does this paper add?**

- F11R/JAM-A homophilic interactions facilitate blood platelets adhesion under flow conditions.
- Cis- and trans-homophilic interactions of platelet F11R/JAM-A take part in thrombus formation, but they are not critical for this process.
