# Supplementary material for: Homophilic Interactions of Platelet F11R/JAM-A with its Surface-Bound Counterpart Facilitate Thrombus Formation
Source: Thromb Haemost. 2025 Apr 18;126(2):179–91. doi: 10.1055/a-2565-9496 (PMC12858303; doi:10.1055/a-2565-9496)
Supplement: Supplementary file 1 — Supplementary Material [file 10-1055-a-2565-9496-s24090469.pdf]

Supplementary Materials S1

Chemicals and Reagents

Buffered sodium citrate or acid-citrate-dextrose/glucose (ACD solution A) was from Becton-Dickinson (Franklin Lakes, New Jersey, United States), blood collection system and hirudin-containing collection tubes were purchased from Sarstedt (Numbrecht, Germany). Apyrase was from Sigma (St. Louis, Missouri, United States). For flow cytometry the following antibodies were used: anti-CD41 antibodies CD41/PE (clone 5B12, DAKO, Agilent Technologies, Santa Clara, California, United States), anti-JAM-A/FITC antibodies (cat 353504, anti-CD321 clone OV-5B8, BioLegend, San Diego, California, United States), J10.4/FITC (cat sc-53623, Santa Cruz Biotechnology, Texas, United States), and their isotypes murine IgG1 (cat 400110, clone MOPC-21, BioLegend, San Diego, California, United States). Coating substrates for adhesion and thrombosis in vitro were human fibrinogen from Calbiochem (Darmstadt, Germany), equine collagen type I from Chrono-log Corp. (Havertown, Pennsylvania, United States), Fc chimeric JAM-A dimer (R&D systems, Minneapolis, Minnesota, United States), JAM-A His-Tag AB276224 Abcam (Cambridge, UK and bovine serum albumin (BSA) obtained from Sigma (St. Louis, Missouri, United States). Cellfix were purchased from Becton-Dickinson (Franklin Lakes, New Jersey, United States). Adhered platelets and thrombi were stained with anti-CD41/PE (R7058, DAKO) and anti- F11R/JAM-A/AlexaFluor488 (clone EP1042Y) antibodies from Abcam (Cambridge, UK), Biolegend OV-5B8 FITC anti-CD321 (BioLegend, San Diego, California, United Sates), phalloidin (AB 176756, Cambridge, UK) and PE Annexin V (556421 BD Pharmingen, San Jose, California, United States). J10.4 antibodies blocking JAM-A dimerization were purchased from Sigma (St. Louis, Missouri, United States) (SAB 4200468) and Santa Cruz Biotechnology sc-53623 (Dallas, Texas, United States). Fragmentation of antibodies to Fc fragments was performed with Pierce murine IgG1 Fab Micro Preparation Kit (Thermo Scientific, Rheinfelden, Germany). Anaesthetics: Sedazine (20 mg/mL xylazine hydrochloride) and ketamine (100 mg/mL ketamine hydrochloride) were obtained from Biowet (Biowet, Pulawy, Poland). Murine JAM-A blocking antibodies BV11 were from Merck Pty Ltd. (Wadeville, Germiston, Republic of South Africa). Ferric (III) chloride and ADP were from Sigma-Aldrich (St. Louis, Missouri, United States). Ferric chloride was placed on filter paper (Whatman, Sanford, Maine, United States). NP-40, deoxycholate, and phenylmethylsulfonyl fluoride (PMSF) were from Sigma, cocktail of protease and phosphatase inhibitors was from Thermo Scientific). Electrophoresis was performed with the use of SDS–PAGE gels (Mini-PROTEAN TGX Gels; Bio-Rad Laboratories, Hercules, California, United States). For western blot nitrocellulose membranes (Bio-Rad Laboratories) were used. Detection was performed with the use of goat anti-Src (cat AF3389, R&D Systems), rabbit anti-phospho-Src (Y416) (cat MAB2685, R&D Systems), rabbit anti-GAPDH (Abcam, Cambridge, GB), Horseradish peroxidase-conjugated anti-goat or anti-rabbit secondary antibodies (Thermo Fisher Scientific) and Pierce ECL Western Blotting Substrate (ThermoFisher Scientific).

Methods

Flow Cytometry

Whole blood was preincubated with 10 or 20 µM ADP for 5 minutes at room temperature (RT). The samples were then diluted 10-fold with phosphate-buffered saline (PBS), labelled with anti-CD41/PE antibodies, anti-JAM-A/FITC antibodies or $IG_1\kappa$/FITC as an isotype control for 15 minutes at RT, and fixed with CellFix for 1 hour at RT. Directly before measurements, the samples were diluted 1:1 with PBS, and the assay was performed, gathering 10,000 CD41/PE-positive events using a FACS Canto II flow cytometer (BD Bioscience, Franklin Lakes, New Jersey, United States). In the gated population of CD41/PE-positive events, the percentage fractions of the platelets positive with regard to JAM-A (above isotype cutoff) were measured. Moreover, the abundance (the number of copies) of given surface membrane markers were presented as a median of fluorescence intensity values (MFI values).

Adhesion to Fibrinogen and F11R/JAM-A under Flow Conditions

Blood platelet adhesion was assessed with the use of VenaFlux platform (Celix, Dublin, Ireland). Channels of Vena8 Endo+ biochip were coated with human fibrinogen (200 µg/mL) overnight at 4°C and blocked with 0.1% BSA for 1 hour at 4°C. Biochip was mounted on a thermo-controlled stage of an inverted AxioVert microscope (Carl Zeiss, Oberkochen, Germany), heating plate maintained the constant temperature of 37°C throughout the experiment. Prior to measurements, the channels were washed with saline. In the experiments involving co-coating of the chips with JAM-A, the chips were coated with 100 µg/mL Fbg and 100 µg/mL Fc-tagged JAM-A. Blood samples were recalcified with $CaCl_2$ in a concentration of 1 mM shortly before measurement. Whole blood was perfused through the channel at 40 dynes/cm^2 (~890 s^{-1}) for 2 minutes. The channel was then perfused with the CellFix for 2 minutes at 5 dynes/cm^2. Next, anti-CD41/PE antibodies were aspired to the channel, and incubated for 30 minutes. The channel was then washed with 100 µL of saline to remove any unbound antibodies. Images of labelled platelets were taken with AxioExaminer microscope using a 20× objective focusing on at least five different ROIs. Area covered by platelets was quantified with the use of Fiji (ImageJ) preceded by segmentation step performed with the use of Ilastik software [19]. Additional experiments were performed to quantify the number of platelets adhering as a function of time. Perfusion conditions were as described above. Adhesion events in real time were recorded. Movie

 © 2025. The Author(s).

sequences were analysed with the use of TrackMate plugin for ImageJ.

Adhesion to Fibrin and F11R/JAM-A under Static Conditions

For the static adhesion study, the surface of Ibidi µ-Slide 15 wells (Ibidi, Martinsried, Germany) was coated overnight with fibrinogen (37.5 µg/mL) at 4°C. After coating, the excess of coating solution was removed, so the reaction with calcified (1 mM) thrombin (1 U/mL over 15 minutes, 37°C) formed a flat net instead of a fully 3-dimensional mesh, which was critical to achieving a uniform plane of focus for platelet observation. Surfaces were additionally treated with recombinant F11R /JAM-A (100 µg/mL) or BSA (0.1%). Platelet-rich plasma was recalcified and incubated over the surfaces for 30 minutes. Next, the wells were washed with Tyrode solution and left to incubate for an additional 30 minutes. Adhered platelets were fixed using CellFix solution, permeabilized with Triton X-100 (0.1%), and stained with Phalloidin for 30 minutes at RT, and excess staining was removed by washing with 100 µL of PBS.

Images were taken in five different fields of view in each well with the use of AxioExaminer microscope (Carl Zeiss, Oberkochen, Germany). Ilastik software was used for machine learning-based classification and quantification of distinct morphological types of blood platelets. To this end, a modified protocol described previously by Pike et al was used [20]. Briefly, a model was trained to differentiate three morphological types of platelets: (1) nonactivated, i.e., round-shaped, devoid of filopodia; (2) presenting at least one filopodium but devoid of lamellipodia, and (3) presenting lamellipodium or fully spread. Images were analysed with the trained model to obtain percentage of platelets presenting each of the phenotypes. Trained Ilastik model is available in ZENODO repository (10.5281/zenodo.13378735).

Confocal Microscopy

Blood platelets incorporated into thrombi in VenaFlux chips channels were stained with anti-CD41/PE and anti-JAM-A/AlexaFluor488 antibodies as described above. Blood platelets adhered to fibrinogen and F11R/JAM-A in VenaFlux chips channels were stained with anti-CD41/PE as described above. F11R/JAM-A staining pattern was visualized using a confocal microscope (Nikon D-Eclipse C1) and analysed with EZ-C1 version 3.6 software (Nikon, Japan).

Fab Enzymatic Preparation

The J10.4 antibodies required a prior removal of their Fc fragments. Enzymatic digestion of the Fc fragment was crucial to remove their potential for platelet activation via the FcγRII, while maintaining the capacity for blocking JAM-A dimerization.

For the preparation of Fabs we used the ficin enzymatic cleaving capacity provided by Pierce murine IgG1 Fab Micro Preparation Kit, in accordance with manufacturers Fab preparation protocol. Successful fragmentation of J10.4 to Fabs of J10.4 was confirmed with the use of SDS-PAGE.

Whole-Blood Impedance Aggregometry

The measurements were performed using Multiplate analyser (Hoffmann-La Roche, Basel, Switzerland) according to the manufacturer's instructions. Briefly, whole blood was preincubated with full J10.4 antibodies or their Fabs for 5 minutes at RT, then 300 µL of blood was transferred into the measurement cell and diluted with 300 µL 0.9% NaCl and preheated to 37°C for another 3 minutes. In selected experiments 0.5 µM ADP (final concentration) was added and platelet aggregation was recorded continuously for 10 minutes. Area under the curve was monitored. All measurements were completed within 3 hours of blood collection.

Thrombus Formation Analysis

Total thrombus formation analysis system (Zacros, Fujimori Kogyo Co. Ltd., Tokyo, Japan) was used to analyze in vitro thrombus formation [21, 22]. In this method the blood is perfused through a capillary coated with a specific substrate. The perfusion pressure that increases as the thrombi buildup is a measure of the capillary occlusion. Reaching of a preset pressure value is considered as a total occlusion of the capillary. The time to the total occlusion as well as area under the curve of pressure changes over time are the quantitative parameters assessed in the method. Depending on the substrate coating the capillary and blood preparation, the assay measures primary or total haemostasis. In the first case, hirudin-anticoagulated blood is perfused through collagen-coated capillary, whereas in the second case citrated and recalcified blood is perfused through collagen and thromboplastin-coated capillary. Blocking agents (IgG, Fabs and soluble F11R/JAM-A His-tagged or Fc-tagged protein) were added 5 minutes prior to perfusion.

In Vivo Models

Total anaesthesia was induced using mix of ketamine (100 mg/kg) and xylazine (10 mg/kg) protocol. The mixture was injected intraperitoneally. Intravenous administration of staining antibodies and tested substances was performed by injection to the retroorbital plexus after confirming full anaesthesia. All surgical procedures were performed using dissecting microscope to aid the operator.

Ferric Chloride-induced Injury

Using sterile sharp/blunt straight-edge scissors, a straight incision was made in the neck midline region from just below the chin to the top of the manubrium sterni. Using dull forceps, the skin on both sides of the incision was spread to the sides with the underlying salivary tissue. Next, the fascia overlying the glandular tissue was exposed and separated in the midline via blunt-dissection to expose the muscular layer. The skin and sternomastoid muscle were collected with a tapered surgical needle (B. Braun surgical, Carretera de Terrassa, Spain), and retracted to the side. Another suture was threaded under the esophagus and the structure was retracted contralaterally to evert and expose carotid artery. With continued blunt-dissection of the fascia with a focus on not damaging vasa vasorum, the right common carotid artery

 © 2025. The Author(s).

and the distal aspect of the carotid artery cephalic are made free of overlying fascia and adjacent nerves.

A strip of a black polyethylene wrap and a strip of filter paper (Whatman, Sanford, Maine, United States) were threaded under the blood vessel to provide isolated field of view and a vehicle for $FeCl_3$ The BV11 antibodies were administered intravenous into the retroorbital plexus. To saturate the filter paper, 3 µL of the 10% ferric chloride was applied. Immediately after administration of $FeCl_3$, the probe of laser Doppler flowmeter was placed directly over the cranial end of the carotid at a height of approximately 1 mm, and data collection ensued. Time of the experiment was measured starting with the application of $FeCl_3$ solution to the paper strip. The measurements were taken for 30 minutes or until reduction of the blood flow to below 10% of the initial value expressed in arbitrary units (LDU) for more than 2 minutes.

Data were collected with the use of a ML191 Blood Flowmeter (ADInstruments, Colorado Springs, Colorado, United States) equipped with a temperature stabilized semiconductor laser diode with emission at 830 ± 10 nm. Measurements were conducted by means of a needle probe connected to the flowmeter through two optic fibers, each of 125 µm in diameter, separated by 300 µm. Maximum laser power at probe tip is 1 mW, according to manufacturer. Doppler shift was analyzed and recalculated to LDF output signal by the flowmeter and transferred to ML870 PowerLab 8/30 data acquisition system (ADInstruments, Colorado Springs, Colorado, United States). All the LDF data were recorded as a function of time by Chart 5 software (ADInstruments, Colorado Springs, Colorado, United States).

Src Phosphorylation

Platelet rich plasma was obtained from whole blood by centrifugation at 200 g for 12 minutes and carefully collected to avoid contamination with red blood cells. Platelets were pelleted by centrifugation at 700 g for 15 minutes and suspended in Tyrode's buffer. Platelet suspension (150×10^3/µL) was poured on wells of a 6-well plate coated with 100 µg/mL fibrinogen. Fab fragments of J10.4 or isotype antibodies were added to final concentration of 50 µg/mL. After 1h incubation at 37°C nonadherent platelets were removed. Adherent platelets were lyzed by incubation with lysis buffer (1% NP40, 0.2% sodium deoxycholate, 150 mM NaCl, 50 mM Tris pH 7.5, 1 mM PMSF, protease, and phosphatase inhibitors cocktail) for 30 minutes at 4°C. Lyzates were centrifuged at 16,000 g for 20 minutes at 4°C. The supernatants were stored at −80°C. To evaluate the level of Src phosphorylation samples with equal total protein content were loaded and separated on SDS–PAGE gels (Mini-PROTEAN TGX Gels; Bio-Rad Laboratories, Hercules, California, United States) and then transferred onto nitrocellulose membranes (Bio-Rad Laboratories). For the analysis of Src levels we used primary antibodies: goat anti-Src (cat AF3389, R&D Systems) and rabbit anti-phospho-Src (Y416) (cat MAB2685, R&D Systems). GAPDH was used as a loading control and detected using rabbit anti-GAPDH (Abcam, Cambridge, GB). Respective HRP-conjugated anti-goat or anti-rabbit secondary antibodies (Thermo Fisher Scientific) were used. The signal was detected by measuring the chemiluminescence with Pierce ECL Western Blotting Substrate (ThermoFisher Scientific). Bands' intensity was quantified with the use of ImageJ. The intensities of the phospho-Src bands were normalized to the respective Src and GAPDH bands.

Statistical Analysis

Data were presented as mean ± standard deviation or median and interquartile range, depending on the normality of data distribution. The Shapiro–Wilk test and Levene's test were used to confirm that the data were normally distributed and homogenous. For normally distributed and homoscedastic variables, the statistical significance of differences between two groups was estimated using the paired or the unpaired Student's *t*-test; for variables that departed from normality and/or variance homogeneity, Mann–Whitney U test was applied instead. To compare differences between more than two groups with a control group, analysis of variance for repeated measures and the Dunnett's post hoc test for multiple comparisons were used. In time-dependent analyses the Kaplan–Meier model was adopted. Comparison of time to occlusion occurrence was performed with the use of log-rank (Mantel–Cox) test. To compare the fractions of samples analysed with the use of T-TAS in which embolization occurs and to compare the fractions of animals that developed occlusion the Fisher's exact test was used. Statistica v. 13.1 (Dell Inc., Tulsa, Ohio, United States), GraphPad Prism v.9 (San Diego, California, United States) were used for statistical calculations and to draw charts.

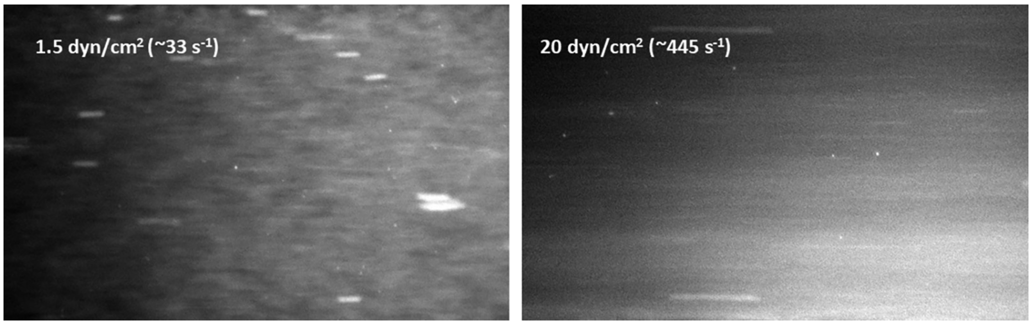

Supplementary Fig. S1 Representative images showing adhesion of fluorescently labelled blood platelets to a surface coated with F11R/JAM-A at two different flow rates. Very few platelets adhered firmly. The effect was similar to that observed at 40 dynes/cm^2 shown in the main body of the manuscript.

 © 2025. The Author(s).

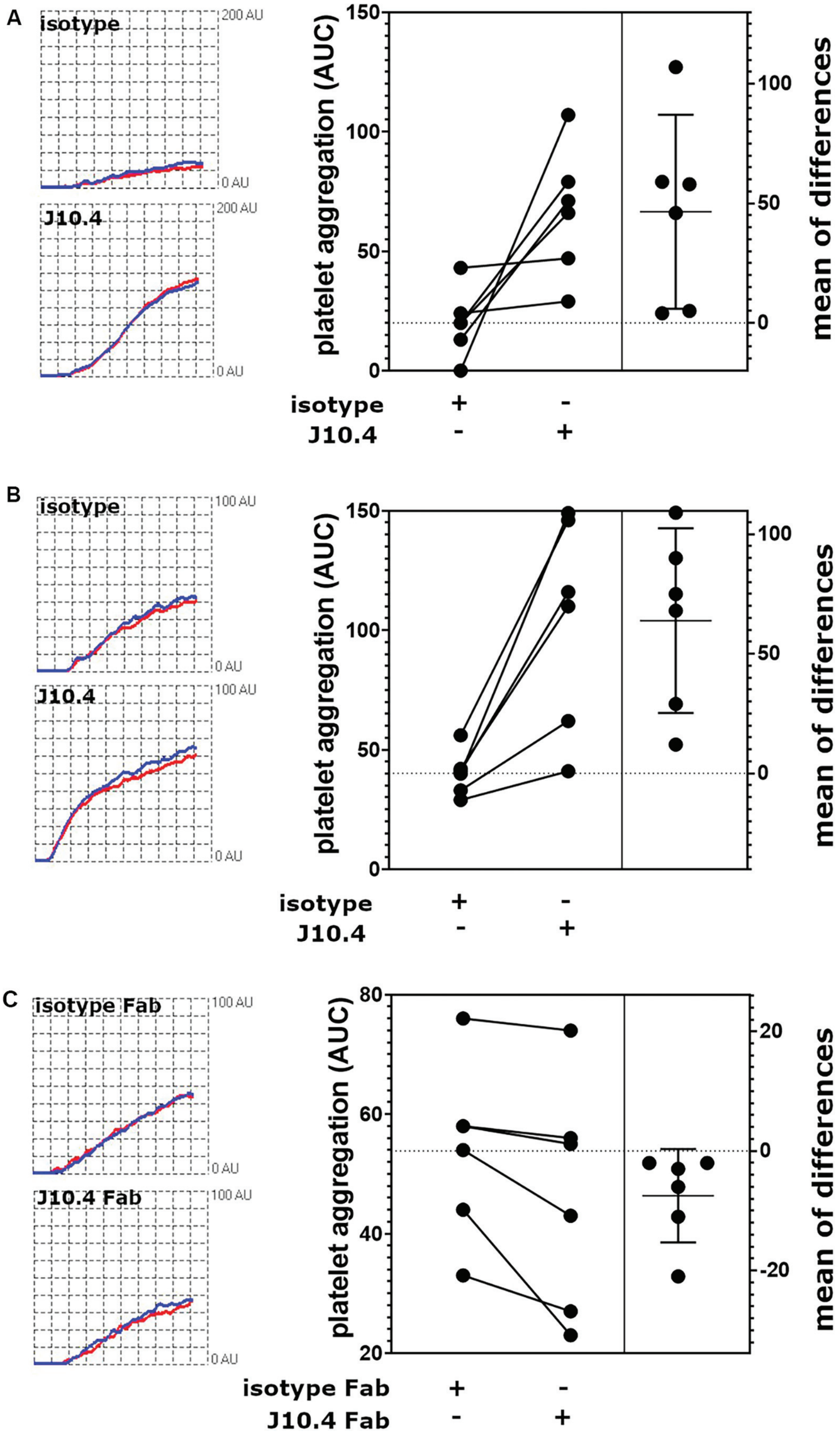

Supplementary Fig. S2 Effects of J10.4 antibodies and their Fab fragments on platelet activation. (**A**) Effects of intact J10.4 antibodies on platelet aggregation compared with isotype antibodies, $P = 0.0321$, paired two-tailed *t*-test, $n = 6$; (**B**) potentiation of platelet response to ADP by J10.4 as compared with isotype antibodies, $P = 0.0081$, paired two-tailed *t*-test, $n = 6$; (**C**) platelet response to ADP in the presence of J10.4 Fab fragments compared with isotype Fab fragments, not significant (n.s.), paired two-tailed *t*-test, $n = 6$. Right panels of the charts a, b and c present differences calculated for each pair of values shown on the left panels (the aggregation in the presence of J10.4 upon subtraction of the aggregation in the presence of isotype).

 © 2025. The Author(s).

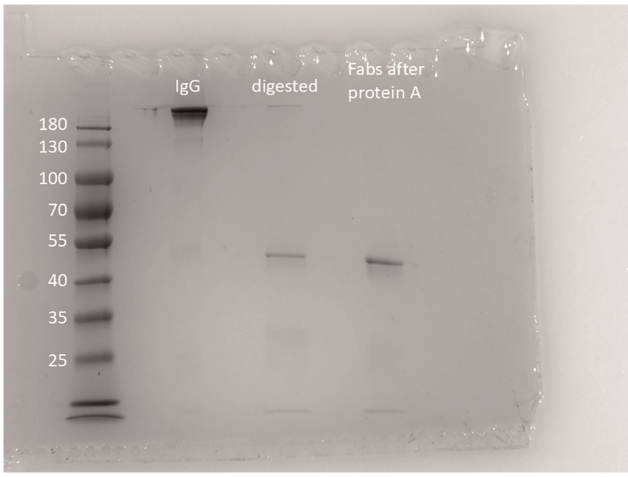

Supplementary Fig. S3 Representative SDS-PAGE gel showing J10.4 fragmentation. IgG—intact J10.4 antibodies, digested—sample after digestion with ficin, Fabs after protein A—sample after incubation with protein A to remove intact IgG. The content of the protein loaded on the lanes was not equalised prior loading. As can be seen, the Fab fragments used in the study are devoid of intact antibodies.

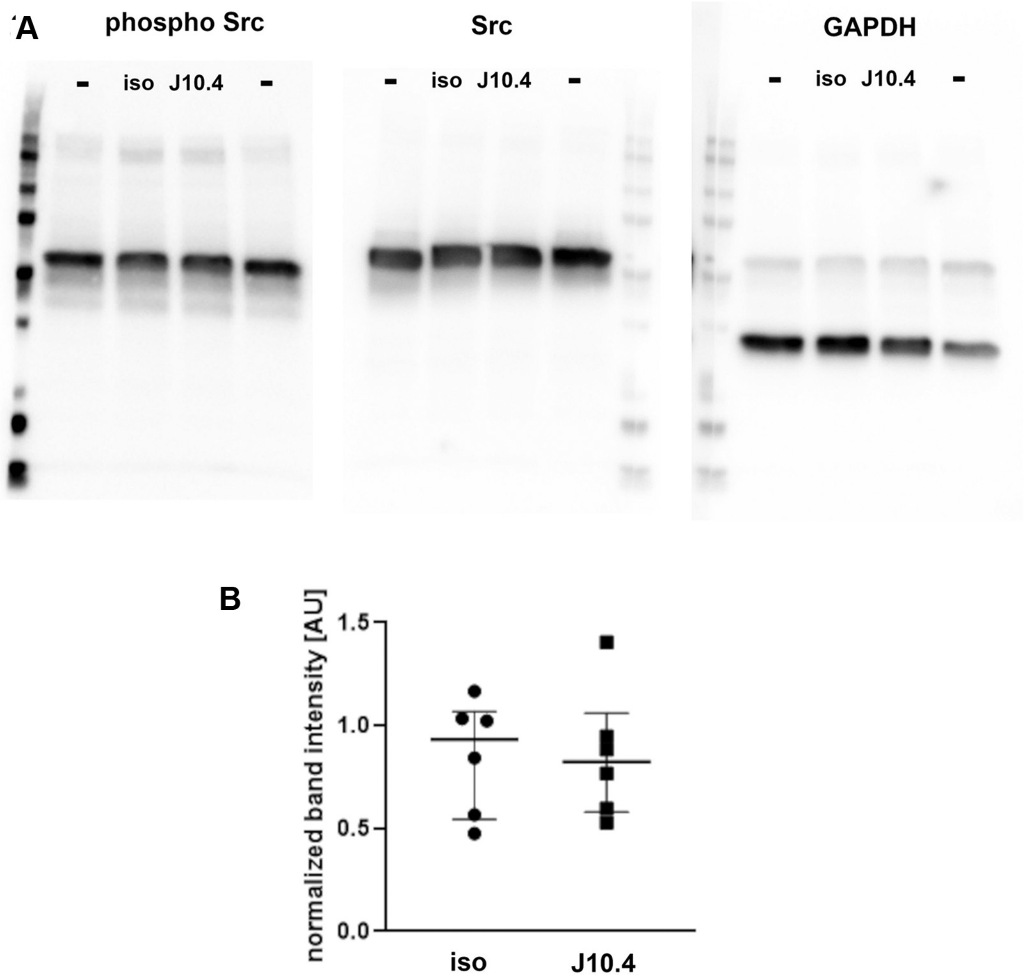

Supplementary Fig. S4 Effect of J10.4 Fabs on Y416 Src phosphorylation in platelets adhering to fibrinogen. (**A**) Representative western blots of Y416 phospho-Src, total Src and GAPDH as an internal control. (**B**) Normalised bands intensity, not significant (n.s.), paired two-tailed *t*-test; $n = 6$.

 © 2025. The Author(s).
